# Characteristics of English Medical Debates

**Published:** 1873-09

**Authors:** 


### CHARACTERISTICS OF ENGLISH MEDICAL DEBATES.

In a letter from London, the writer speaking of the discussions in the Pathological Society, says :—"The whole tendency of our English societies is, however, to discourage orations ; there is always observable a nervous desire to avoid oratory on the part of the speakers, and a great impatience of anything approaching to it among the auditors. Short, dry statements of unadorned facts and arguments are what is expected, and nothing else is tolerated. Hence we have not among us more than two or three orators ; and these are always careful to repress their capabilities in speaking at the medical societies, and only display their powers elsewhere. Sir James Paget is a born orator ; so are Sir Thomas Watson and Sir William Gull and Mr. Savory ; but they are rarely heard at the societies in more than a few sentences, delivered in a studiously subdued monotone and with a deliberately passive manner which excludes all gesture. When occasionally a foreign orator, such as M. Ricord or Demarquay, or M. Labbe′ (recently), has addressed English meetings of medical men, as at the last meeting of the British Medical Association, it has been curious to observe the half-startled, half-amused expression of their listeners, as they poured forth their orations with animated gestures, in high-pitched notes, and with the artifices of interrogation, response, protest, sarcasm and voluble argument which belong to the trained speaker who expects his auditors to follow him by the hour together."

